# Exploring the Guessing‐Game Experimental Paradigm: Inferences From Closed‐ Versus Open‐Ended Semantic Space

**DOI:** 10.1111/cogs.70199

**Published:** 2026-03-23

**Authors:** Svetlana Kuleshova, Aleksandra Ćwiek, Stefan Hartmann, Michael Pleyer, Marta Sibierska, Marek Placiński, Johan Blomberg, Przemysław Żywiczyński, Sławomir Wacewicz

**Affiliations:** ^1^ Center for Language Evolution Studies Nicolaus Copernicus University in Toruń; ^2^ Institute of Advanced Studies Nicolaus Copernicus University in Toruń; ^3^ ArScAn‐Équipe AnTET (UMR 7041), CNRS Université Paris Nanterre; ^4^ Leibniz‐Zentrum Allgemeine Sprachwissenschaft (ZAS); ^5^ German Department Heinrich Heine University Düsseldorf; ^6^ Centre for Languages and Literature Lund University

**Keywords:** Experimental semiotics, Semantic space, Ecological validity, Understanding, Bayesian hierarchical modeling, Conceptual replication

## Abstract

How we measure success in signal comprehension experiments fundamentally shapes our conclusions. Two recent studies have demonstrated that humans can guess the meanings of novel vocalizations and ape gestures above chance when selecting from limited alternatives. We replicated both experiments using open‐ended responses instead of multiple choice. For the vocalization data, where participants provided single‐word or short‐phrase responses, we systematically compared three evaluation methods applied to the same responses: exact matching, graded similarity ratings, and computational semantic similarity. For the gesture data, we applied graded similarity ratings. Each evaluation method revealed a different semantic landscape. Participants’ success was very low when measured by exact matching, moderate by similarity ratings, and substantially greater by computational measures, which capture broader thematic connections. Despite these differences, a consistent pattern emerged across both datasets and all evaluation methods: success was determined primarily by properties of the signals (their semantic category and degree of transparency) rather than individual participant abilities. Participants often reliably distinguished broad categories (actions vs. objects, animals vs. artifacts) but rarely identified specific concepts—and these distinct patterns only became visible through a combination of evaluation methods. In sum, our results partly align with the original studies yet also diverge in ways conducive to different conclusions about naïve humans’ ability to understand novel vocalizations or ape gestures. We show that closed‐ versus open‐ended response formats, and different evaluation scales, function as complementary research tools rather than competing approaches. Each reveals different aspects of how humans navigate semantic space when interpreting novel signals. Experimental and evaluation designs are, therefore, not a technical detail but a theoretical choice about which semantic relationships we seek to expose.

## 1 Introduction

Research in language evolution has seen a surge of interest in the *motivatedness* of signals, that is, the degree to which there is a principled connection between form and meaning of a signal. Arguably, the most central and well‐studied form of such motivatedness is iconicity, which refers to a relationship of resemblance between form and meaning (Winter, Woodin, & Perlman, 2026). A wealth of research has shown that iconicity is much more pervasive in language than previously assumed (see, e.g., Dingemanse, Blasi, Lupyan, Christiansen, & Monaghan, 2015; Fischer et al., 2026), challenging the classic Saussurean notion of the arbitrariness of the sign. The question of to what extent interlocutors can communicate using motivated, and especially iconic signs has, therefore, become a key issue in language evolution research and beyond. There is increasing evidence that iconic signs in different modalities can convey a much broader array of meanings than previously assumed (see Ćwiek et al., 2021, and the references cited therein), lending support to the hypothesis that in language evolution, iconic signs may have served as a crucial bridge from presymbolic communication to fully fledged language.

Two recent experimental studies (Ćwiek et al., 2021; Graham & Hobaiter, 2023) have demonstrated that humans can guess the meanings of novel vocalizations and ape gestures, respectively, when choosing from limited alternatives. Ćwiek et al. (2021) showed that listeners from 28 diverse linguistic and cultural backgrounds correctly identified meanings of nonlinguistic vocalizations at rates reliably exceeding chance. These vocalizations were originally crowdsourced through the “Vocal Iconicity Challenge” (Perlman & Lupyan, 2018), where participants produced nonlinguistic sounds for 30 concepts spanning diverse semantic categories: animate entities (e.g., snake, child), inanimate entities (e.g., water, fruit), properties (e.g., small, dull), actions (e.g., cut, gather), demonstratives (this, that), and quantifiers (one, many). Ćwiek et al. tested whether naive listeners could identify these vocalizations’ intended meanings. In their cross‐cultural online study, participants listened to vocalizations and selected meanings from six alternatives; in on‐site studies, participants selected from 12 pictured referents (entities only). Participants could replay vocalizations as many times as needed. Results showed above‐chance identification across all tested languages and cultural contexts, suggesting robust cross‐cultural iconicity effects.

Graham and Hobaiter (2023) demonstrated similar above‐chance performance with great ape gestures. Participants watched 20 videos of bonobos and chimpanzees gesturing to each other in naturalistic contexts and guessed gestural meanings from four alternatives. The study included 10 gestures total (e.g., “feed me,” “groom me”), which means that the participants watched every gesture being performed by both species. Five of these gestures had alternative meanings in different contexts, with the contextually inappropriate meaning included among response options. Videos could be replayed as needed and were accompanied by a “bonobo‐bot” cartoon highlighting the gesture in question. The experiment tested two conditions: one providing contextual information about what happened immediately before the gesture, and one without such context. Participants performed above chance in both conditions, with context having only a marginal positive effect.

Both studies employed closed‐ended semantic spaces through multiple‐choice paradigms. This design offers clear methodological advantages: chance baselines are mathematically defined (1/*n* alternatives), enabling straightforward statistical hypothesis testing; cross‐linguistic comparison is simplified through standardized response options; and forced‐choice formats are easier to implement across diverse populations. These studies made important contributions by demonstrating that iconic vocalizations and ape gesture meanings are guessable above chance—establishing that some form−meaning transparency exists in these signals.

However, the closed‐ended design also introduces a fundamental interpretative question: to what extent do these results reveal general properties of signal transparency versus artifacts of the design and evaluation decisions? Success in multiple‐choice tasks partly depends on semantic crowding among alternatives (cf. Discussion); for example, a participant who distinguishes *animal* from *object* succeeds if those are the only options, even without identifying the specific animal. Additionally, the evaluation paradigm varies widely across broader iconicity and gesture literature—from binary forced‐choice (Köhler, 1929; Ramachandran & Hubbard, 2001) to magnitude estimation (Thompson & Estes, 2011) to *n*‐ended production (e.g., Perlman, Dale, & Lupyan, 2015 with 10‐ and 18‐alternative forced‐choice)—, yet systematic investigations of how evaluation design shapes conclusions remain limited. However, we argue that examining how the choice of the evaluation scale influences the results can contribute significantly to a better understanding of how and to what extent people are able to reliably guess the intended meanings of iconic vocalizations and gestures. After all, when trying to figure out what a given signal means, it clearly makes a difference whether one can choose from a range of options, and which options are available to choose from.

We, therefore, conducted conceptual replications (Hudson, 2023) of both studies, replacing the closed‐ended, multiple‐choice design with an open‐ended, free‐text response. This choice allowed us to test the comprehension of the vocalizations and gestures without any context restrictions, which is an extremely rare methodological choice due to the associated complexity in logistics and evaluation. To address that, we employed a multievaluation approach: we assessed the same free‐text data using three complementary measures (for vocalizations: binary exact matching, ordinal similarity judgments, and computational cosine similarity; for gestures: ordinal similarity judgments). This design allows us to examine how the evaluation scale choice shapes which semantic patterns become visible in the data.

Our results lead us to formulate four key points. First, methodologically, we demonstrate that the same experiment conducted within open‐ and closed‐ended semantic space paradigms may lead to different results and invite markedly different conclusions. We show that the success of humans in guessing the meanings of novel vocalizations and ape gestures largely seems to derive from the closed‐ended design; (however, we also show that even in an open‐ended design, participants’ guesses are far from random, as the responses are often at least somewhat conceptually close to the target meaning, demonstrating these effects are not simply artifacts of multiple‐choice constraints). Second, in line with the previous point, we show that evaluation scale choice is not merely a technical detail but a theoretical decision determining which semantic relationships become observable. Different evaluation methods applied to identical response data expose fundamentally different “semantic landscapes.” Third, we advance and nuance prior findings by showing that success typically occurs at the level of semantic domains rather than specific concepts—a distinction only visible with the evaluation granularity afforded by open‐ended designs. Certain semantic categories (e.g., actions associated with distinctive sounds like *sleep/snoring*) are inherently more guessable than others. And finally, our Bayesian hierarchical modeling reveals that stimulus properties (iconicity, semantic category), rather than participant‐level cognitive variation, overwhelmingly determine success, suggesting transparency effects are robust properties of signal‐meaning mappings and not products of individual differences.

## 2 Closed‐ versus open‐ended semantic spaces: Theoretical and methodological considerations

The two studies we replicate—Ćwiek et al. (2021) and Graham and Hobaiter (2023)—share the aim of testing whether humans can infer, or “understand,” the meanings of novel signals, yet they differ in the mechanisms they implicitly or explicitly invoke to explain success. In the case of vocalizations, Ćwiek et al. (2021) interpret above‐chance guessing as evidence that listeners rely on iconicity, that is, resemblance‐based mappings between signal form and meaning. Certain sounds, such as those imitating snoring or chewing, may provide perceptual cues that facilitate inference. Graham and Hobaiter (2023) treat the question of which mechanisms explain human performance in interpreting ape gestures as unresolved. They raise several explanatory possibilities that might explain humans’ above‐chance responses, such as sharing the same social goals and body plans, general intelligence, or a shared action‐understanding mechanism between humans and great apes based on the resemblance of gestures and their intended behavioral response.

These studies jointly raise a broader question: to what extent can we interpret successful guessing in a constrained, multiple‐choice setting as informative of real‐world communicative situations and of higher‐order communicative capacities, such as understanding? Similar concerns have been voiced in experimental semiotics and language‐evolution research, where task‐specific success is often interpreted as evidence for communicative competence (Fay, Garrod, & Roberts, 2008; Galantucci, 2005; Galantucci & Garrod, 2011). If performance primarily reflects recognition of form–meaning correspondences or other perceptual heuristics, then apparent comprehension may result from sensitivity to surface cues rather than from grasping communicative intent (Dingemanse et al., 2015; Harnad, 1990)—with an extreme example being the *Clever Hans* effect: In the early 1900s, a horse named Clever Hans appeared to be able to solve mathematical tasks, but it could be demonstrated that he only reacted to the bodily cues that his trainer unwittingly gave him (Pfungst, 1911/1965; Sebeok & Rosenthal, 1981).

For this reason, both the everyday use and the theoretical accounts of *understanding* in cognitive science and philosophy typically go beyond mere recognition or guessing accuracy. Understanding is often characterized as involving manipulation of mental representations (Wilkenfeld, 2013), making deep associations (Boden, 2001), mental simulation of perceived actions (e.g., Zwaan & Madden, 2005), or participatory sense‐making in interaction (e.g., Di Paolo, Cuffari, & De Jaegher, 2018). Related discussions in second‐language learning similarly distinguish shallow lexical inferencing from deeper semantic understanding (Hu & Nassaji, 2014; Nation, 2022), with lexical coverage research consistently showing that 80–90% word knowledge is required for minimal adequate text comprehension (van Zeeland & Schmitt, 2012; Durbahn, Rodgers, Macis, & Peters, 2024; Laufer & Ravenhorst‐Kalovski, 2010; Webb, 2021). These perspectives suggest that guessing above chance in constrained contexts, while informative, may not alone warrant strong claims about understanding.

Our study takes a complementary methodological approach to the two studies under discussion. We manipulate a single design parameter—the structure of the semantic space—to examine whether participants converge on similar interpretations when the semantic space is unrestricted.

## 3 Methods

### 3.1 Participants

Two hundred participants were recruited via Prolific for the vocalization experiment, and 202 for the ape gesture study. All participants gave informed consent before they started the experiment. In both studies, most participants reported English as their first language (vocalizations: 53, apes: 66), followed by Portuguese (vocalizations: 41, apes: 28), Spanish (vocalizations: 27, apes: 33) and Polish (vocalizations: 19, apes: 26).^1^ Twenty‐one of the participants reported to have studied nonhuman primates, such as in a university course. A separate analysis of their data showed that they did not perform significantly better than the participants without previous experience in that domain, which is why we decided not to remove the data of those individuals.

Traditional power analysis was not applicable to our study for three reasons. First, we conducted conceptual replications with fundamental design changes (closed‐ vs. open‐ended semantic space), meaning original effect sizes did not transfer. Second, we introduced novel outcome measures—particularly graded similarity judgments and distributional semantic similarity—for which no prior effect‐size estimates existed. Third, our vocalization analysis employed Bayesian estimation methods where sample size planning focuses on the precision of parameter estimates rather than frequentist power (Kruschke, 2015).

We, therefore, determined sample size pragmatically: we matched the per‐stimulus response counts from the original studies (enabling direct comparison) while ensuring sufficient data for precise posterior estimation in our hierarchical models. This yielded *N*≈200 participants per experiment, providing narrow credible intervals around stimulus‐level estimates and robust estimation of random‐effect structures.

In the post‐experiment survey, we used the same questions as Ćwiek et al. (2021), adding two 5‐point items on perceived task complexity and the extent to which participants felt they had guessed the meanings correctly. Nearly all participants reported no hearing disabilities (99.5%), most completed the task at home (94%), and audio device use was balanced (55% headphones; 44% speakers), while input devices were mostly computers (88.5%). Self‐reported toughness showed limited variability (*M* = 3.25, *SD* = 0.94; 73% between 3 and 4 on a 5‐point scale), and the extent to which the participants thought they guessed correctly also clustered narrowly around the midpoint (*M* = 2.62, *SD* = 0.78; 82.5% between 2 and 3 on a 5‐point scale). Due to this restricted variance, neither measure was included as a predictor in the statistical models.

In the post‐experiment survey for the chimpanzee gesture study, we also asked participants if they guessed what the experiment was about; out of the 180 participants who provided a free‐text answer to this question, 138 assumed, in one way or another, that the experiment was about interpreting the “body language” (this term was used in 16 responses) of chimpanzees and bonobos, with many assuming that the experiment was about figuring out how ape gestures are perceived from a human perspective. (Interestingly, no less than 46 respondents used the term *understand* in their response.)

### 3.2 Materials

We used the same stimuli as in the original studies. For the vocalization experiment, we used the 90 nonlinguistic iconic vocalizations from Perlman and Lupyan's (2018) “Vocal Iconicity Challenge,” as employed in Ćwiek et al. (2021). These target concepts spanned multiple semantic categories: animate entities (e.g., *snake*, *child*), inanimate entities (e.g., *water*, *fruit*), actions (e.g., *sleep*, *eat*, *cut*), properties (e.g., *small*, *bad*), demonstratives (e.g., *this*, *that*), and quantifiers (*one*, *many*). For the ape gesture experiment, we used the 40 video clips from Graham and Hobaiter (2023): 10 distinct gesture types (e.g., of meanings *Groom me*, *Give me that food*, *Move away from me*), each performed by both a bonobo and a chimpanzee in their natural habitats.

### 3.3 Procedure

Our conceptual replication reproduced the design of the original experiments except for the response format: we replaced the multiple‐choice paradigm with open‐ended free‐text responses. Both experiments were implemented in Labvanced (Finger, Goeke, Diekamp, Standvoß, & König, 2017), and participants were recruited through Prolific (eligibility criteria: age 18+, fluent in English).

**Fig. 1:**
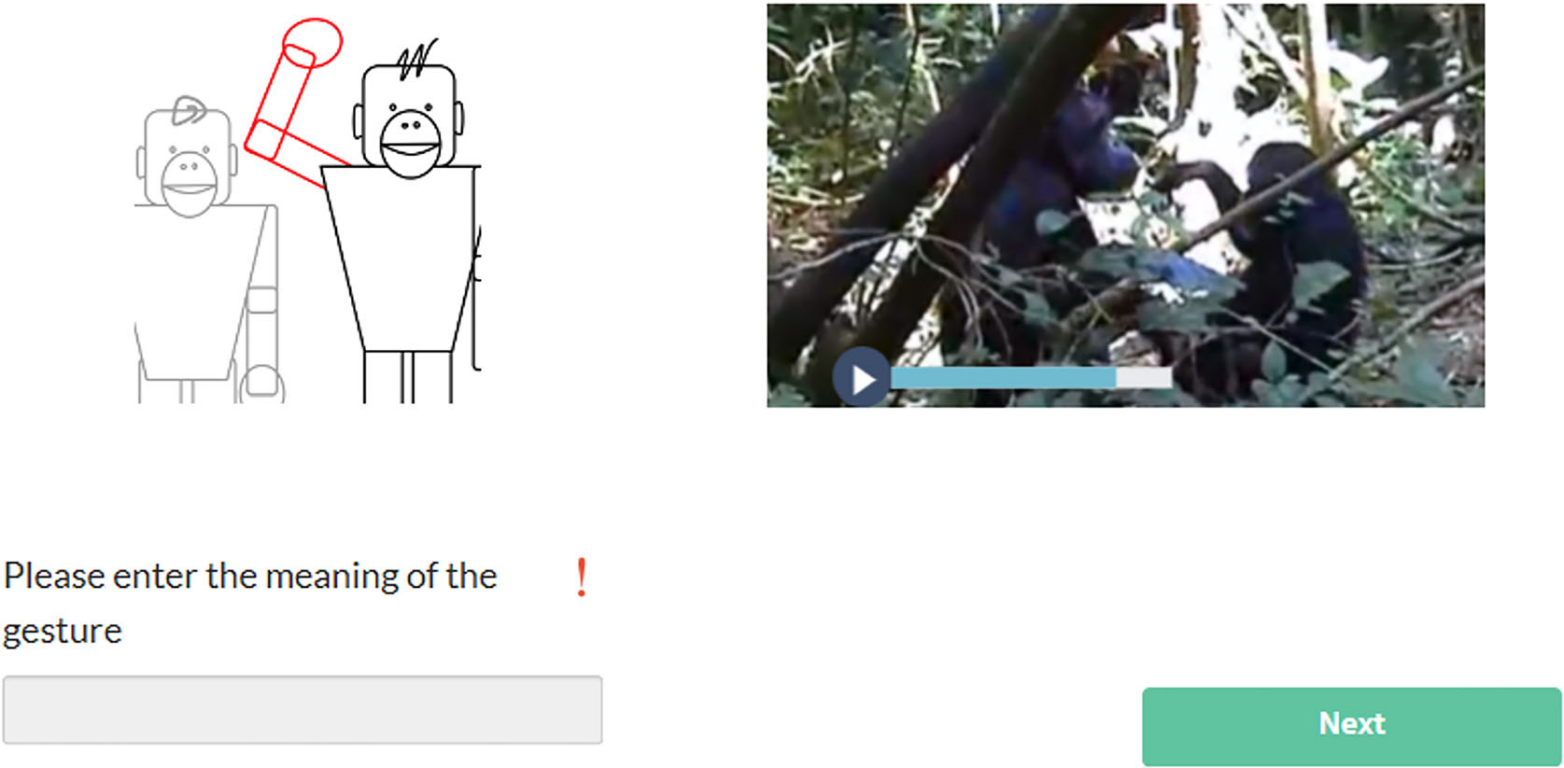
Example of the setup of the replication of the Graham and Hobaiter (2023) experiment.

In the vocalization experiment, participants listened to all 90 vocalizations in randomized order and typed what they thought each vocalization meant. In the gesture experiment, participants watched all 20 videos in randomized order and typed what they thought each gesture meant. In both experiments, stimuli could be replayed as many times as needed, but responses could not be submitted without experiencing the stimulus at least once. In the gesture experiment, each video first played the gesture in slow‐motion and then at normal speed. A “bonobo‐bot” (schematic drawing) appeared alongside each gesture video to help identify the target gesture. Following Graham and Hobaiter (2023), we implemented two between‐subjects conditions for gestures: participants in the context condition received written descriptions of what happened immediately before the gesture, while those in the no‐context condition saw only the video. We also followed Graham and Hobaiter's procedure in subdividing each group into two subgroups, in which participants watched different sets of videos. Fig. 1 shows an example of the user interface.

Before starting, participants completed a brief survey indicating their native language, input/output devices, whether they were in a calm environment, and (for the gesture experiment) whether they had studied nonhuman primates or animals in a university course. For the vocalization experiment, participants also indicated whether they spoke a dialect of their native language, which other foreign languages they spoke, and whether they had any hearing disabilities. At the end of both experiments, participants rated how difficult they found the task, evaluated their own responses, and guessed the aim of the experiment. Unlike Ćwiek et al. (2021), who tested 25 languages online, we conducted our experiment in English only. Ethical approval for the study was obtained on April 26, 2024, from the ethics board of the Faculty of Arts and Humanities of Heinrich Heine University Düsseldorf.

### 3.4 Data coding

All free text responses were independently coded using a 4‐point ordinal scale: 0 = meaning is very different (not related or only very distantly related); 1 = meaning is only partly similar; 2 = meaning is very similar but not identical; 3 = same meaning and same phrasing (but, e.g., differently inflected forms of the same lexeme were acceptable, see the example in Table 1). Table 1 illustrates this scheme with examples for the vocalization *cut*. In this table, *cutting* is rated as 3 because it is the same word (albeit in an inflected form). *Chopping* is rated as 2 because it is not identical to *cutting*, but it is very similar in terms of action itself, and the versatility of the action, for example, *chop the onion* versus *chop wood*. Similar to *cut*, it typically implies a change of state; it is more specific than *saw* in that the change‐of‐state (the separation of material) is quick and sudden, as indicated by the OED definition of *chop*: “To cut with a quick and heavy blow.”^2^ *Sawing*, on the other hand, is rated as 1 even though the meaning is partly similar. Among other aspects, one major difference to *cut* and *chop* is that atelic readings (“using a saw”—without necessarily fully cutting the material) seem to be more common. Finally, *scratching* is rated as 0. Even though it may involve partly similar movements and actions, it can be considered semantically very distant from cutting (no separation of material).

**Table 1:** Coding scheme with examples for the vocalization cut

Score	Meaning of the score	Example response (target: *cut*)
0	Meaning is very different	Scratching
1	Meaning is only partly similar	Sawing
2	Meaning is very similar but not identical	Chopping
3	Same meaning and same phrasing	Cutting

We used a strict annotation scheme requiring exact or near‐exact phrasing for the highest ratings, which provides an unambiguous operationalization that minimizes subjective interpretation. While this stringency means high ratings (2−3) are relatively rare, the 4‐point scale captures meaningful gradations: responses can be unrelated (0), semantically adjacent (1), close approximations (2), or exact matches (3). Importantly, differences in part of speech (e.g., *drinking* for target *water*, but not *drinking* for target *drink*) were coded as 0, as these represent different conceptual categories. The same coding scheme was applied to both experiments for consistency and comparability.

Four coders (one external, three authors) independently rated all responses. The external coder rated all responses; the three author‐coders each rated one‐third, ensuring each response received two independent ratings. Inter‐rater reliability was assessed using Krippendorff's alpha for ordinal data (Krippendorff, 1980). Overall agreement was low‐to‐moderate (mean Krippendorff's α = 0.58, standard error = 0.03^3^), with slightly higher agreement for vocalizations than gestures. Disagreements were resolved through team discussion, with all mismatches reaching a unanimous resolution.^4^ As an example of such a mismatch, we can look at the answer *teenager* for a target concept *child* (vocalization experiment). Potential ratings were 1 and 0. After the discussion, the coders agreed on 1, because a teenager, by not being a full adult, is still partially similar to a child and should, therefore, be treated as semantically adjacent. Most disagreements involved distinguishing between codes 0 and 1 (unrelated vs. partly similar), suggesting coders agreed on the presence or absence of semantic relatedness but sometimes differed on its degree.

For the vocalization data, we supplemented human coding with a semantic similarity measure to address limitations of the strict coding scheme. Specifically, we computed cosine similarity between target concepts and participant responses using ConceptNet Numberbatch^5^ embeddings (Speer, Chin & Havasi, 2017), a graph‐structured semantic database that represents relations between natural language words, integrating multiple lexical resources (WordNet, Wiktionary, crowdsourced data, word embeddings). For multiword responses, we extracted the head noun/verb. This distributional approach captures broader semantic relatedness (e.g., *water‐drinking*) that the ordinal coding scheme treats as unrelated due to category boundaries. Together, the three measures—binary exact match, ordinal similarity, and cosine similarity—provide complementary perspectives on semantic success at different levels of granularity.

We applied all three evaluation methods to the vocalization data, but only ordinal similarity judgments to the gesture data. This asymmetry reflects response structure: vocalization responses were predominantly single words (e.g., *sleep*, *snoring*), enabling direct comparison via exact matching and word embeddings. Gesture responses were typically longer descriptive phrases or sentences (e.g., *the ape wants the other one to come closer*), which do not map straightforwardly onto single‐concept embeddings. The ordinal rating scheme, accommodating semantic relatedness independent of linguistic surface form, proved most appropriate for cross‐dataset comparison.

### 3.5 Statistical analysis

For the statistical analysis, we focused on the vocalization guessing experiment, since all three evaluation scales were available for these data. We analyzed the data using Bayesian hierarchical models implemented in the brms package (Bürkner, 2017) with the cmdstanr backend in R (version 2.37.0). We fitted separate models for each of the three outcome measures: (1) binary accuracy was modeled using a Bernoulli distribution with a logit link; (2) ordinal judged similarity (0–3) was modeled using a cumulative logit model; (3) cosine similarity (continuous, [0,1]) was modeled using a Beta distribution after min−max normalization (which mapped the observed range including negative values to [0,1]) followed by epsilon‐transformation (which avoids exact boundary values of 0 and 1 required for Beta distribution).

To enable direct comparison of stimulus‐level similarity across measurement approaches, all measures were transformed to a common [0, 1] scale, though the timing and method of transformation differed by measure type. For binary guessing accuracy (Bernoulli model), predictions are naturally bounded on [0, 1]. For ordinal similarity judgments (cumulative model, 0–3 scale), we computed expected values from posterior predictive distributions and normalized by dividing by 3, yielding values on [0, 1]. For ConceptNet cosine similarities, however, post‐model transformation was insufficient due to the compressed empirical range of observed values (original scale: min = −0.157, max = 1.00; rescaled to 0–1: min = 0.42, max = 1.0). Fitting a Beta regression to this compressed scale would have resulted in posteriors calibrated to a distribution artificially shifted toward high similarity, making cross‐measure comparison invalid. Therefore, we applied min−max normalization to cosine values prior to modeling: x_scaled = (x − min(x)) / (max(x) − min(x)). This transformation preserved the relative distances between all stimulus pairs (Spearman ρ = 0.988 for stimulus‐level estimates) while mapping the observed range onto [0, 1], ensuring that a value of 0.5 represents the midpoint of observed similarity in each measure. Following the transformation, all three measures were analyzed with their respective model families (Bernoulli, cumulative ordinal, Beta), and stimulus‐level random effects were extracted and compared on the common [0, 1] scale, representing each stimulus pair's position within the observed similarity range for each measure.

All models included varying intercepts for participants and stimuli to account for individual differences and item‐level variation. We used weakly informative priors: Normal (0, 5) for intercepts and Student‐*t* (3, 0, 5) for standard deviations. Models were run with four chains, 8000 iterations (4000 warmup), and convergence was assessed using $\hat{R}$ < 1.01 and effective sample size > 400.

## 4 Results

We present our results in three parts. First, we describe the distribution of responses across our three evaluation measures to establish their empirical landscape (Section 4.1). Second, we report Bayesian hierarchical model estimates for the vocalization experiment, showing how participant and stimulus variation patterns differ across measures (Section 4.2). Third, we demonstrate how evaluation scale choice fundamentally shapes conclusions about communicative success (Section 4.3). Finally, we compare our results to the original studies (Section 4.4).

### 4.1 Response distributions across evaluation measures

**Fig. 2:**
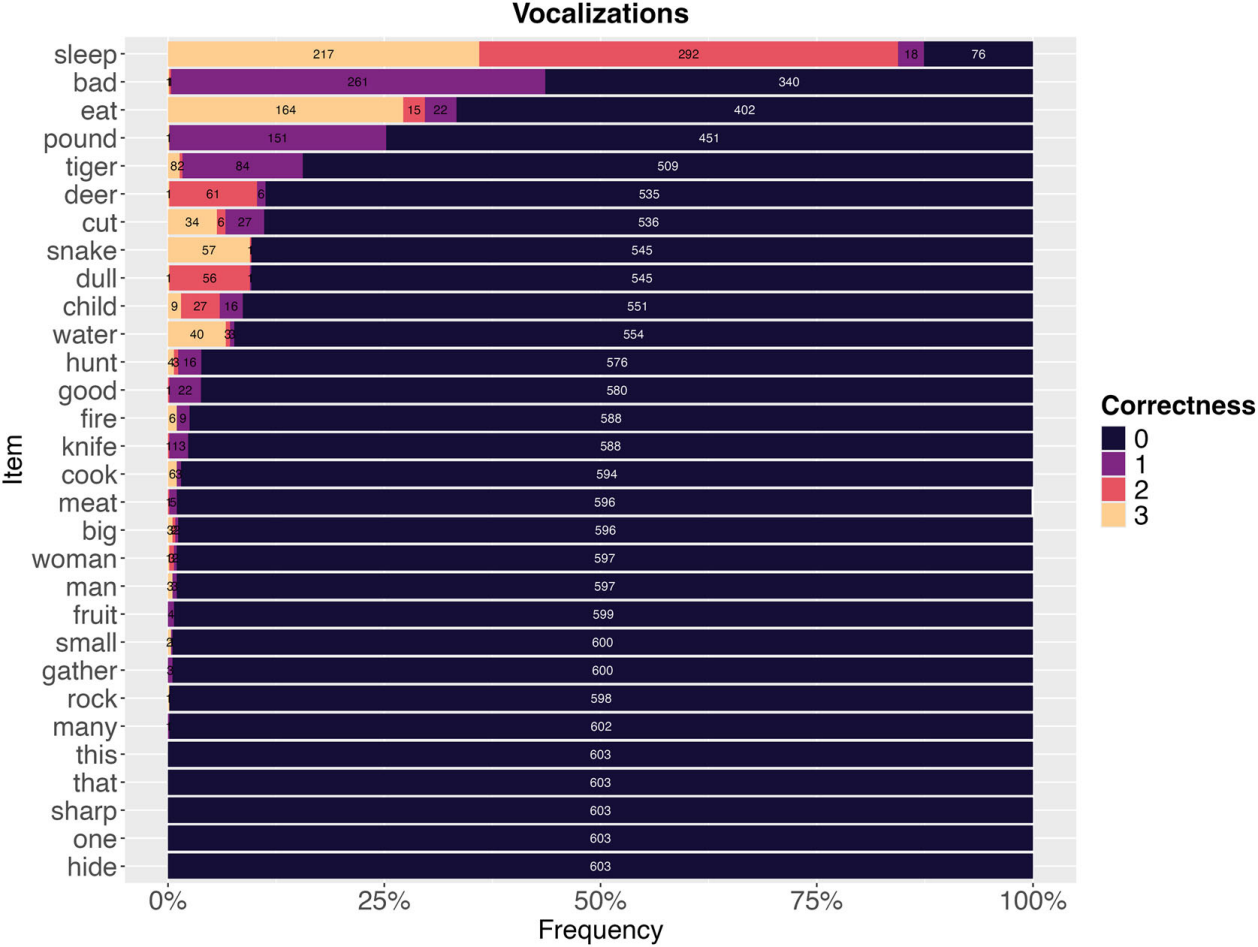
Distribution of ordinal similarity ratings (0−3 scale) for each vocalization. Most responses were rated as completely dissimilar (0) to target meanings, though a few concepts—particularly sleep, bad, and eat—showed higher proportions of partial or complete matches.

For the vocalization experiment, across 90 vocalizations and 200 participants, we collected 27,279 responses. Fig. 2 shows the distribution of ordinal similarity ratings for each vocalization, revealing that most responses (87.6%) were rated as completely dissimilar (0) to their target concepts. Only a small proportion received higher ratings: 7.8% were partly similar (1), 3.8% were very similar (2), and 0.8% were exact matches (3). However, substantial variation existed across concepts. The vocalization *sleep* showed the highest success, with 32.1% responses rated as similar or exact (codes 2–3)—largely driven by participants recognizing it as *snore* or *snoring* (249 of 603 responses), which was coded as very similar (2). The concept *eat* also performed well, with participants frequently producing the exact target (164 responses). In contrast, abstract concepts like demonstratives (*this*, *that*) and quantifiers (*one*, *many*) received almost exclusively dissimilar ratings (0).

**Fig. 3:**
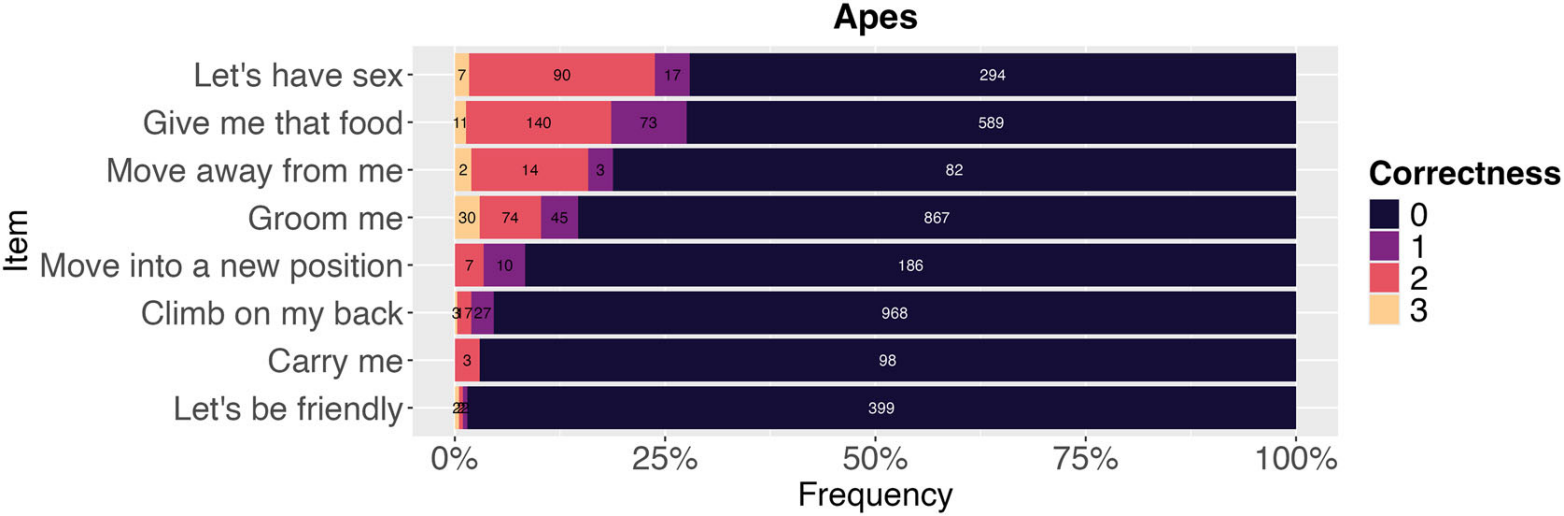
Distribution of ordinal similarity ratings (0−3 scale) for each ape gesture meaning. As with vocalizations, most responses showed no similarity to target meanings, with modest success for a subset of gesture types.

For the ape gesture experiment, across eight different gestures and 202 respondents, we collected 4062 responses. As Fig. 3 shows, the gesture meanings *Let's have sex*, *Give me that food*, and *Move away from me* elicited the highest proportions of similar responses, though success rates remained modest. Contextual information (descriptions of what happened before the gesture) did not improve guessing accuracy, contrary to the marginal effect observed in the original study.

**Fig. 4:**
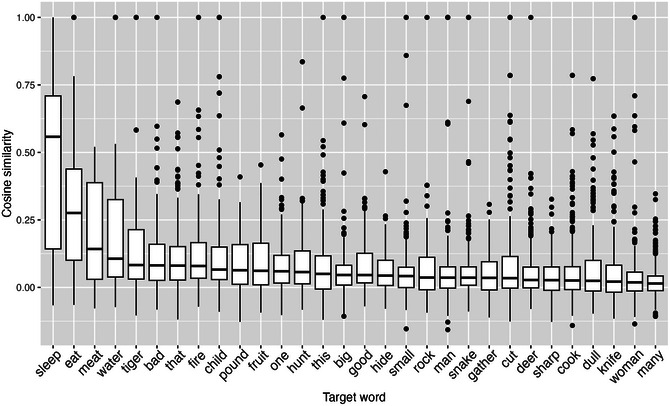
Cosine similarity between target concepts and participant responses using ConceptNet embeddings. Unlike binary or ordinal measures, distributional semantics reveals that many responses occupied semantically adjacent regions even when they did not match target words exactly.

Fig. 4 presents automated semantic similarity (cosine distance) between participant responses and the target concept using ConceptNet embeddings. This distributional measure showed a different pattern: while exact lexical matches remained rare, many responses occupied nearby regions of semantic space. The vocalization *sleep* again ranked highest (*M* = 0.784), reflecting both exact matches (*sleep/sleeping*) and the semantically adjacent *snore/snoring*. Notably, even concepts with zero exact matches showed nontrivial cosine similarities, indicating that participants often accessed the correct semantic domain without producing the target word. For instance, responses like *drinking* for target *water* (coded as dissimilar under ordinal ratings due to part‐of‐speech and conceptual mismatch) nonetheless showed semantic proximity in distributional space.

Table 2 summarizes descriptive statistics across all three measures. Binary coding (exact matches only) showed *M* = 0.048 (*SD* = 0.215, Median = 0), indicating 4.8% exact matches. Ordinal similarity ratings yielded *M* = 0.29 (*SD* = 0.77, Median = 0) on the 0–3 scale; normalized to [0,1], this corresponds to *M* = 0.097 (*SD* = 0.257, Median = 0). Cosine similarity (rescaled to [0,1]) showed *M* = 0.588 (*SD* = 0.122, Median = 0.59); after min−max normalization for cross‐measure comparability, *M* = 0.287 (*SD* = 0.21, Median = 0.24).

**Table 2:** Descriptive statistics for three evaluation measures across all vocalizations

Measure	Scale	Mean	SD	Median
Binary accuracy	0−1	0.05	0.22	0
Ordinal similarity (raw)	0−3	0.29	0.77	0
Ordinal similarity (normalized)	0−1	0.10	0.26	0
Cosine similarity (rescaled)	0−1	0.59	0.12	0.59
Cosine similarity (min−max normalized)	0−1	0.29	0.21	0.24

Performance varied substantially across semantic categories. Actions showed the highest success across all measures: binary *M* = 0.139, normalized ordinal *M* = 0.242, and cosine *M* = 0.652. Demonstratives (*this*, *that*) and quantifiers (*one*, *many*) showed zero exact matches, near‐zero ordinal ratings, and low cosine similarities (*M* = 0.154−0.230). Abstract properties (adjectives) showed binary *M* = 0.002−0.004, normalized ordinal *M* = 0.0002−0.058, and cosine *M* = 0.153−0.285. Among nouns, animate entities (animals) outperformed inanimate objects and people across all measures.

The three evaluation measures showed strong positive correlations (Table 3), indicating they capture overlapping but distinct aspects of semantic similarity. Cosine similarity and normalized ordinal judgments correlated most strongly (*r* = .833, 95% HDI [0.826, 0.839]), while binary and cosine measures showed the weakest correlation (*r* = .682, 95% HDI [0.670, 0.693]). This pattern suggests that graded similarity measures align more closely with each other than with strict binary classification.

**Table 3:** Correlations between evaluation measures of the vocalization data

Pair	*r*	95% HDI lower	95% HDI upper	BF_10_
Cosine ∼ Ordinal	.83	0.83	0.84	>10^3^ ^0^
Cosine ∼ Binary	.68	0.67	0.70	>10^3^ ^0^
Ordinal ∼ Binary	.80	0.79	0.81	>10^3^ ^0^

*Note*. Pearson correlations with Bayesian posterior estimates and 95% highest density intervals. BF_10_ indicates the Bayes factor for correlation ≠ 0. Ordinal judgments were normalized to a 0–1 scale for comparability.

### 4.2 Bayesian model results

We fitted three separate Bayesian hierarchical models to characterize how well participants’ free‐text responses matched target concepts across different evaluation approaches. Each model included random intercepts for participants (*N* = 200) and stimulus concepts (*N* = 30) to account for individual differences in performance and systematic variation in concept guessability. All models converged successfully.

#### 4.2.1 Binary accuracy model

We modeled whether responses were exact matches using a Bernoulli likelihood with a logit link. The population‐average probability of an exact match was very low: on the logit scale, the intercept was −7.11 (95% CI: [−8.65, −5.75]), which corresponds to only a 0.08% probability of success for an average concept and average participant. However, substantial heterogeneity existed across both participants (*SD* = 1.50, 95% CI: [1.29, 1.73]) and stimulus concepts (*SD* = 3.48, 95% CI: [2.43, 5.02]). The larger standard deviation for concepts indicates that some concepts were far more guessable than others, with differences spanning several orders of magnitude in probability.

#### 4.2.2 Ordinal model (human judged)

We modeled human‐rated similarity ratings (0 = completely different, 3 = identical meaning) using a cumulative logit model. The three threshold parameters were 3.90 (95% CI: [2.80, 5.03]), 4.88 (95% CI: [3.78, 6.01]), and 6.19 (95% CI: [5.09, 7.32]) on the logit scale. These thresholds reflect the boundaries between adjacent rating categories and indicate that responses were predominantly in the lower similarity categories. Converting to the original 0–3 scale and normalizing to [0, 1], the expected similarity across all observations averaged approximately 0.33, indicating that most responses captured only partial semantic overlap with targets. Participant variability (*SD* = 0.67, 95% CI: [0.58, 0.76]) was moderate, while stimulus concepts again showed considerable heterogeneity (*SD* = 3.08, 95% CI: [2.28, 4.23]).

#### 4.2.3 Cosine similarity model

We modeled ConceptNet semantic similarity scores (rescaled and min−max normalized to [0, 1]) using a Beta regression with a logit link. The population‐level intercept on the logit scale was −0.53 (95% CI: [−0.86, −0.22]), corresponding to a mean cosine similarity of approximately 0.37 on the probability scale. The precision parameter φ = 2.75 (95% CI: [2.71, 2.79]) indicates moderate dispersion in the responses around their means. Participant‐level variation (*SD* = 0.35, 95% CI: [0.31, 0.39]) was smaller than in the other models, suggesting that automated semantic similarity captured relatively stable individual differences. As with the other measures, concepts varied substantially (*SD* = 0.88, 95% CI: [0.68, 1.16]), confirming that some target meanings produced responses much closer to the target in semantic space than others.

Across all three models, a consistent pattern emerged: stimulus‐level variation substantially exceeded participant‐level variation. This indicates that the guessability of novel vocalizations was primarily determined by properties of the signals themselves (iconicity, semantic domain, acoustic features) rather than individual cognitive differences. The extremely low intercepts for binary accuracy and below‐midpoint intercept for cosine similarity confirm that, in absolute terms, semantic guessing success was limited when the response space was unrestricted—yet relative differences between stimuli were substantial and systematic.

### 4.3 Evaluation scale shapes conclusions about communicative success

**Fig. 5:**
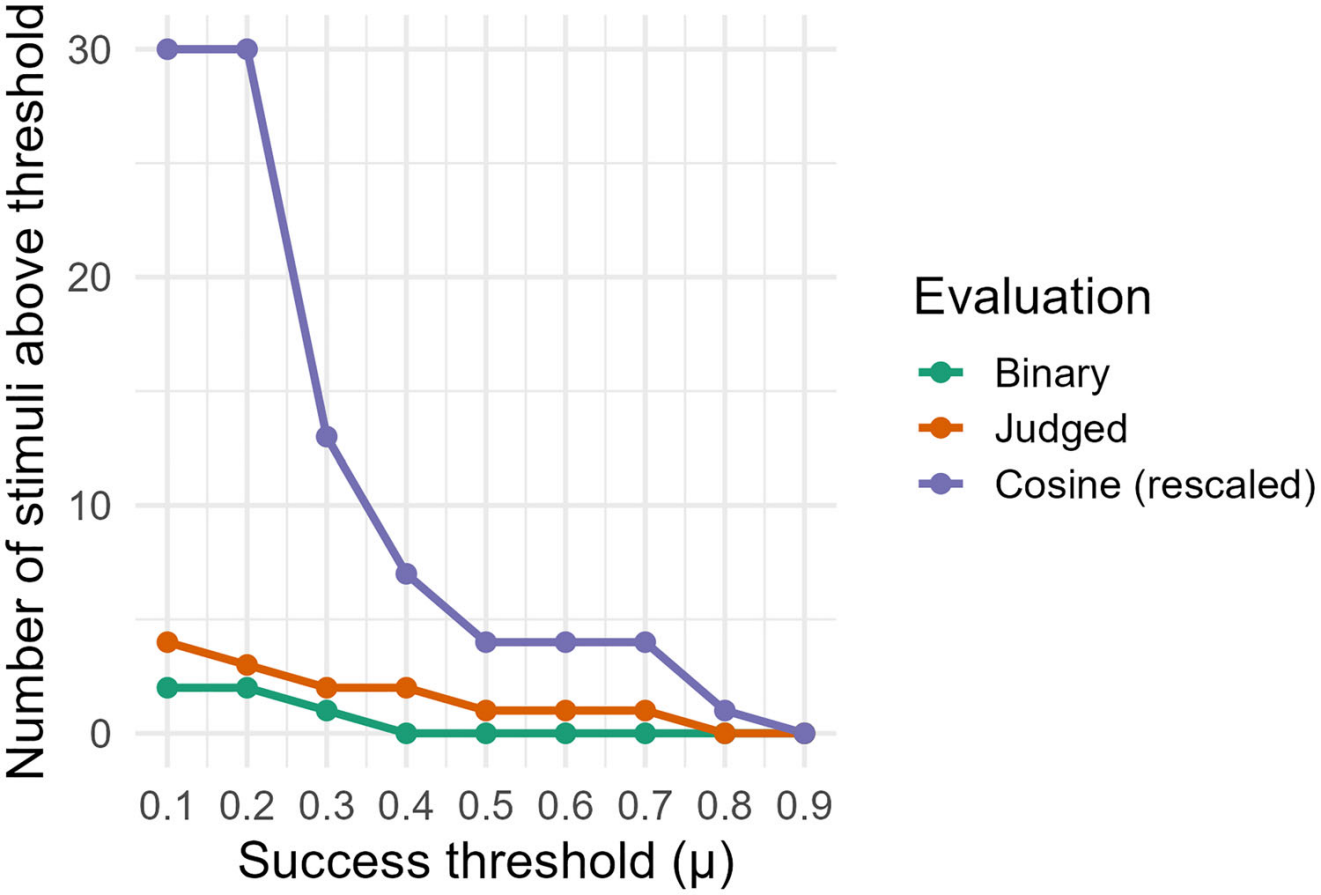
Number of stimulus concepts exceeding success thresholds by the evaluation method. Cosine similarity (purple) identifies substantially more concepts as successful at all threshold levels compared to binary accuracy (green) and judged similarity (orange). The three methods converge only at the most stringent threshold (μ = 0.9), where no concepts succeed.

The three evaluation approaches produced markedly different assessments of communicative success. Fig. 5 illustrates how the choice of metric determines which concepts qualify as “successfully communicated.” At a lenient threshold (μ = 0.1), cosine similarity classified all 30 concepts as showing some success, while binary and judged similarity approaches identified fewer than five concepts. This divergence persisted across threshold levels: at μ = 0.3, cosine similarity still identified 13 successful concepts, whereas binary and judged metrics dropped to nearly zero. Only at the strictest threshold (μ = 0.9) did all three approaches converge in identifying no successful concepts.

**Fig. 6:**
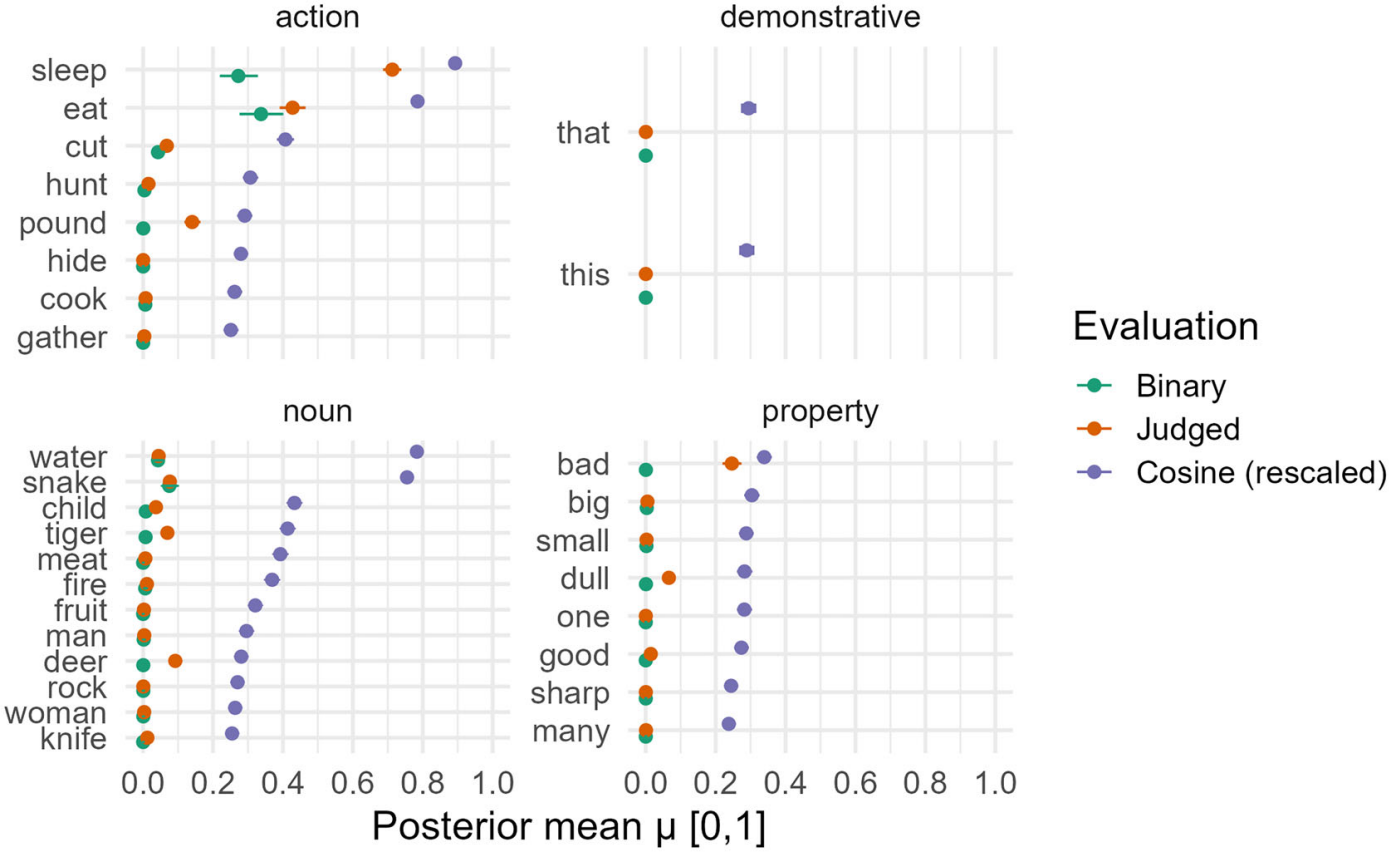
Stimulus‐level posterior estimates by semantic category and evaluation method. Points show posterior means with 95% credible intervals (horizontal error bars) for binary accuracy (green), judged similarity (orange), and cosine similarity (purple). Most error bars are too narrow to be visible, reflecting high posterior precision from the hierarchical models. Cosine similarity consistently produces higher estimates than the other methods, particularly for action verbs. Error bars reflect posterior uncertainty.

Fig. 6 shows stimulus‐level posterior estimates organized by semantic category. Cosine similarity systematically assigned higher performance estimates than binary or ordinal approaches across all categories. For the action concepts *sleep* and *eat*, binary accuracy reached 0.2−0.3 (the highest across all stimuli), ordinal similarity reached approximately 0.5−0.7, and cosine similarity reached 0.8−0.9. The property *bad* showed near‐zero binary accuracy but moderate ordinal ratings (≈0.3) and substantial cosine similarity (≈0.5). Demonstratives and quantifiers showed consistently low performance across all measures.

This pattern reveals that the three measures function as progressively inclusive lenses on semantic success. Binary accuracy (exact matches) sets the strictest criterion, identifying only concepts with strong iconic affordances (actions with characteristic sounds). Ordinal similarity (human judgments) captures a middle ground, crediting partial matches and semantically adjacent responses. Cosine similarity (distributional semantics) exposes the broadest pattern, showing that participants often accessed correct semantic domains (e.g., FOOD, ACTIONS) even when specific lexical targets differed. Critically, these are not measurement errors producing discrepant results—they are complementary theoretical lenses exposing different aspects of semantic guessing. Evaluation scale choice is, thus, not a methodological detail but a substantive theoretical decision about which level of semantic granularity counts as “success.”

### 4.4 Comparison of the results

We obtained a complex and interesting pattern of results that partly converges with and further nuances the original studies, while also showing important differences. Consistent with Ćwiek et al. (2021), the vocalization *sleep* showed the highest guessability in our replication, and the rank ordering of concepts (which performed best vs. worst) largely aligned with the original study, though absolute success rates were lower in our open‐ended design. In Ćwiek et al., actions were guessed at the highest rate, followed by animate entities, with demonstratives the lowest, and our ordinal and cosine measures partially replicated this pattern, while binary accuracy barely exposed any well‐guessable concepts at all.

For ape gestures, Graham and Hobaiter (2023) found that context had a marginal positive effect for ambiguous gestures; in contrast, we found no context effect in our replication. The best‐guessed gestures differed somewhat between studies: the original identified *Climb on my back/Move into a new position*, *Give me that food*, and *Groom me*, while our replication identified *Let's have sex*, *Give me that food*, and *Move away from me*.

Importantly, while rank orderings showed some consistency, absolute success rates were substantially lower in our open‐ended paradigm than in the original multiple‐choice designs. This demonstrates that the closed‐ended format provided a scaffolding—participants could discriminate among a set of alternatives more successfully than they could generate target meanings from unrestricted semantic space.

## 5 Discussion

A major point of our paper concerns how the experimental paradigm shapes the inferences we can make about signal comprehension. Our results show that closed‐ended and open‐ended designs are complementary methods that reveal different aspects of semantic transparency.

Closed‐ended designs are widely used in experimental semiotics and comprehension research (e.g., Garrod, Fay, Lee, Oberlander, & MacLeod, 2007; Krauss & Weinheimer, 1966; Fay, Arbib & Garrod, 2013; Fay, Ellison & Garrod, 2014; Zlatev, Wacewicz, Zywiczynski, & Weijer, 2017; Żywiczyński et al., 2021, see Delliponti et al., 2023 for an overview). Using them offers substantial advantages: precise measurements, clear operationalization of success as proportion of correct responses, straightforward statistical testing with defined chance baselines, and logistical efficiency. These designs excel at hypothesis testing, cross‐cultural comparison, and establishing whether discrimination occurs above chance. Open‐ended designs combined with multiscale evaluation require more resources as they necessitate decisions about instruction framing as well as multiple coding approaches, and they entail substantial annotation workload. However, they reveal semantic structure that remains invisible in forced‐choice paradigms. They expose gradations of similarity, domain‐level clustering, and which semantic relationships participants spontaneously access. Most importantly, they avoid a key limitation of closed designs: *semantic crowding effects*.

The choice between closed and open formats critically interacts with semantic crowding (Wilson, Ellison, & Fay, 2014). Closed designs vary in difficulty depending on three parameters: (1) *size*—the number of distractor items alongside the target, easily controlled through chance baselines; (2) dynamism—whether options remain static or change across trials, engaging working memory (Cowan, 2001; Miller, 1956); and (3) most critically, crowding—the semantic distance between target and distractors. A gesture meaning “give me food” is easy to distinguish from “let's watch Netflix” but much harder from “give me fruit.” Conversely, participants who distinguish “animal” from “object” succeed in closed designs even without identifying specific animals—this is useful for some research questions but obscures fine‐grained semantic structure (see also Crivelli, Russell, Jarillo, & Fernández‐Dols, 2017; Gendron, Roberson, van der Vyver, & Barrett, 2014; Russell, 1994 for similar discussion about facial expressions).

As our study shows in the open‐ended semantic space paradigm, evaluation scale choice fundamentally shapes which semantic relationships become visible in experimental data. Applying binary exact matching, ordinal similarity judgments, and computational cosine similarity to the same vocalization responses revealed that each measure exposes a distinct “semantic landscape.”

Binary exact matching showed extremely low success rates (baseline probability ∼0.08%), with only three concepts (*sleep*, *eat*, *bad*) exceeding 10% exact matches. Ordinal human judgments revealed a different pattern: while exact matches remained rare, 12.4% of responses showed some semantic relatedness (codes 1–3), indicating participants often accessed semantically adjacent concepts without producing target words. Cosine similarity captured the broadest patterns, showing that responses frequently occupied nearby regions of semantic space even when lexically distinct from targets (e.g., *drinking* for *water*, *snoring* for *sleep*).

Critically, Bayesian hierarchical modeling revealed consistent patterns across all three measures: stimulus‐level variation (e.g., *SD* = 3.48 for binary accuracy) substantially exceeded participant‐level variation (*SD* = 1.50), demonstrating that guessability is primarily determined by signal properties—semantic category, iconicity—rather than individual cognitive differences. This pattern held across binary, ordinal, and continuous measures, suggesting that transparency effects are robust properties of signal‐meaning mappings rather than products of individual variation or measurement artifacts.

These findings demonstrate that evaluation scale choice is not a technical detail but a theoretical decision about which semantic relationships we privilege. Binary measures highlight exact lexical convergence (rare but unambiguous), ordinal measures capture graduated relatedness (revealing domain‐level clustering), while distributional measures expose broader thematic connections. Each serves distinct research purposes, and together they provide complementary perspectives onto semantic structure that no single measure alone could reveal.

This has important implications for what conclusions can be drawn from guessing game research tasks. If we were to use success in a multiple‐choice paradigm as the only evaluation method, this could lead to the interpretation that such success means that participants “understand” the signals whose meaning they have to guess. Conversely, if we were to use failure in an unconstrained open‐ended semantic space guessing task as the only evaluation method, this could lead to the interpretation that such failure means that participants “do not understand” the signals whose meanings they have to guess. Here, we show that these approaches are not exclusive, and that neither of these options represents the one correct approach to elucidating what participants “understand.” Instead, as we show, different evaluation methods are complementary and offer different insights into how people navigate semantic space when making inferences about the meaning of signals.

Our results also extend findings from prior iconicity research (e.g., Ćwiek et al., 2021; Perlman et al., 2015). Consistent with the original studies, we found that certain vocalizations—particularly *sleep* and *eat*—showed substantially higher guessability than others, and this pattern held across all three evaluation measures. They succeeded because snoring is a culturally widespread sound‐concept pairing; *eat* succeeded because chewing sounds are perceptually distinctive. In contrast, abstract concepts (demonstratives like *this/that*, quantifiers like *one/many*) and concepts lacking distinctive acoustic signatures (most properties and many objects) showed near‐zero success across all measures. This pattern aligns with broader iconicity research showing that resemblance‐based mapping is context‐dependent. Iconicity can facilitate the emergence of novel signs, but it often gives way to more conventionalized and less transparent forms as communicative systems evolve (Edmiston, Perlman, & Lupyan, 2018). In this view, iconicity offers an initial scaffold for meaning creation, not a stable mechanism for comprehension. This is exactly what our study showed: that even though the participants were able to guess larger semantic domains in the vocalization replication, it would probably be insufficient to pass or understand a concrete message.

Together, our findings highlight a basic conclusion that closed and open designs answer different questions. Closed designs ask, “Can participants discriminate targets from specific alternatives?” Open designs ask, “What semantic space do participants spontaneously navigate?”

This brings us to ecological validity, understood as the relevance to real‐life contexts. Importantly, *neither closed‐ nor open‐ended semantic spaces perfectly represent natural communication*. They represent opposite extremes of a spectrum: closed designs provide too much constraint (limited alternatives guide interpretation), while fully open designs provide too little (unrestricted vocabulary, no pragmatic context).

In natural communication, the semantic space is simultaneously constrained and open. Physical environment, shared context, common ground, social situation, and pragmatic inference all constrain interpretation—but crucially, the *mind* remains unrestricted in how it generates possible meanings.

Our study demonstrates one extreme (fully open, no context); prior studies demonstrated another (fully closed, limited alternatives). Future research should explore methodological approaches that better approximate this natural middle ground. One possibility would be to systematically manipulate semantic crowding within closed designs, varying the similarity between alternatives from minimal to maximal. This would expose not just whether participants can guess above chance, but which levels of semantic granularity iconic vocal or gestural cues can support.

Another approach would involve expanding the semantic space gradually within a single study. Participants might begin with binary forced choice, then face four alternatives, then eight (for different stimuli), with researchers tracking where performance degrades. Alternatively, the semantic space could be gradually restricted.^6^ Such designs would also allow researchers to isolate the specific contribution of semantic crowding versus absolute difficulty, since the same target concepts could be tested across different choice‐set sizes.

Perhaps most ambitiously, researchers could move from symbol manipulation tasks toward action‐based operationalizations of comprehension. In this approach, understanding would be demonstrated not by selecting or producing verbal labels, but by acting appropriately in response to signals. Galantucci's (2005) coordination games in experimental semiotics exemplify this: participants create novel symbols to help partners navigate spaces or coordinate actions, with success measured by behavioral outcomes rather than verbal agreement. Applied to gesture comprehension, this might involve participants responding to an ape gesture meaning “give me food” by actually offering food items, nonfood items, or withholding objects entirely. Success would be defined not by saying the words “give me food” but by acting as the signaler intended. Such designs would constitute understanding in a pragmatic sense—as acting appropriately in an environment—and would likely capture something closer to real‐world communication than verbal guessing tasks, whether closed or open.

These methodological considerations ultimately reinforce our central arguments: that experimental and evaluation designs are a theoretical choice determining which aspects of semantic transparency become observable. The extent to which participants successfully interpret signals reflects not only the expressive affordances of those signals (their iconicity, their pragmatic clarity, their perceptual distinctiveness) but also the structure of the task itself—how constrained the response space is, how much context is provided, and whether success is measured verbally or behaviorally. Our findings thus highlight both the cognitive and experimental limits of resemblance as a basis for signal comprehension. Resemblance cues can support interpretation, but primarily at the level of semantic domains and primarily when combined with appropriate evaluation methods that make such domain‐level convergence visible. Moving forward, the field would benefit from explicitly matching evaluation paradigms to research questions, recognizing that closed designs, open designs, and hybrid approaches each illuminate different facets of how humans navigate meaning in communication.

## 6 Conclusion

Our conceptual replications provide converging evidence with prior findings while revealing important boundaries. Like Ćwiek et al. (2021) and Graham and Hobaiter (2023), we found above‐chance success for certain signals—but largely at the domain level (actions vs. object, animate vs. inanimate), rarely for specific target concepts. Unlike the original studies, many concepts showed near‐zero success across all measures, and context did not improve ape gesture recognition. In that sense, the original studies’ claims about humans “understanding” novel vocalizations or ape gestures are likely to be overstated. These patterns only became visible through open‐ended, multiscale evaluation, demonstrating that methodological choices determine which semantic relationships we can observe.

Evaluation scale choice is a theoretical decision, not a technical detail. By applying three complementary methods to the same open‐ended responses, we demonstrated that different measures expose fundamentally different “semantic landscapes”: binary accuracy reveals exact lexical convergence (rare), ordinal judgments capture graduated relatedness (moderate), and semantic similarity measures expose domain‐level clustering (broader). Critically, across all measures, individual concepts rather than participants determined success—iconicity and transparency are properties of specific signals, not products of individual cognitive variation.

Most importantly, we show that closed‐ended and open‐ended semantic spaces are complementary research tools, not competing approaches. Neither represents natural communication, where minds navigate constrained‐but‐open semantic spaces using context, common ground, and pragmatic inference. Closed designs excel at testing discrimination; open designs reveal semantic structure. The central insight of our work is that evaluation paradigms act as distinct lenses, each exposing different facets of signal transparency. Therefore, researchers must explicitly align methodological choices with theoretical questions about which semantic relationships matter for their inquiry.

## Conflict of interest

The authors declare that they have no known competing financial interests or personal relationships that could have appeared to influence the work reported in this paper.

## Data Availability

The data and analysis scripts are available at https://doi.org/10.17605/OSF.IO/GRHYC.

## Notes

1 We used ChatGPT 4 (OpenAI 2023) for obtaining the ISO codes of the first languages from the participants’ free‐text entries in order to standardize the free‐text responses. We manually checked the results.

2
https://www.oed.com/dictionary/chop_v1?tab=meaning_and_use#9564885 (last checked 20/02/2026).

3 As the coding was done by different combinations of annotators, we calculated IAA separately for each of the 35 sheets provided to the annotators. The lowest IAA value was 0.43, the highest one was 0.93.

4 A reviewer correctly points out that resolving annotation conflicts in group discussions has major drawbacks, as there can be many incentives for settling on a particular option that are motivated by nonscientific factors. We would still argue that it is a useful strategy as long as one remains aware of its limitations and, perhaps even more crucially, as long as the decision process is well‐documented. This is why we make the list of mismatches available in the Supplementary Material (folder data_annotated/helpers/mismatches; see Section “Data Availability Statement”).

5
https://github.com/commonsense/conceptnet‐numberbatch/ (last checked 17/02/2026).

6 Thanks to a reviewer for this suggestion.
